# Character Strengths as “Values in Action”: Linking Character Strengths With Values Theory – An Exploratory Study of the Case of Gratitude and Self-Transcendence

**DOI:** 10.3389/fpsyg.2021.576189

**Published:** 2021-02-04

**Authors:** Shiri Lavy, Maya Benish-Weisman

**Affiliations:** ^1^Department of Leadership and Policy in Education, University of Haifa, Haifa, Israel; ^2^Paul Baerwald School of Social Work and Social Welfare, The Hebrew University of Jerusalem, Jerusalem, Israel

**Keywords:** values, character strengths, social outcomes, prosocial behavior, social acceptance, gratitude

## Abstract

Character strengths are widely studied positive traits considered to be “values in action,” reflecting morally valued virtues. They are hypothesized to serve as pathways to the manifestation of values in life for the benefit of individuals and societies. However, there is surprisingly limited theoretical writing and empirical research on the expected links of character strengths with specific values [e.g., as defined by Schwartz (1992)] or on character strengths as the pathway for behavioral and social manifestations of these values. In this paper, we delineate theoretical links between the two theories and outline their implications. We then provide an initial empirical examination of a specific character strength – gratitude, as a pathway from Schwartz’s self-transcendence values (self-reported) to prosocial behavior and peer acceptance (rated by peers), in two samples of adolescents (9th grade and 11th grade). The findings indicated that most pathways were significant, providing initial support for the theoretical model. However, in one of the samples, the indirect path from self-transcendence values to prosocial behavior was only marginally significant. Taken together, the findings point to the need for further research on the role of character strengths in creating a pathway from values to various social outcomes.

## Introduction

Character strengths constitute a family of positive traits (Peterson and Seligman, 2004) reflecting routes to morally valued virtues (Dahlsgaard et al., 2005). They are often considered to be “values in action” (VIA; e.g., Park and Peterson, 2006), as each strength is related to the application of a certain virtue and reflects psychological mechanisms fostering its practice (Peterson and Seligman, 2004). The 24 character strengths are thus hypothesized to serve as the mechanisms enabling the behavioral practice of moral virtues in everyday life for the benefit of the individuals who practice them and others in their social environment (Peterson and Seligman, 2004).

**FIGURE 1:**
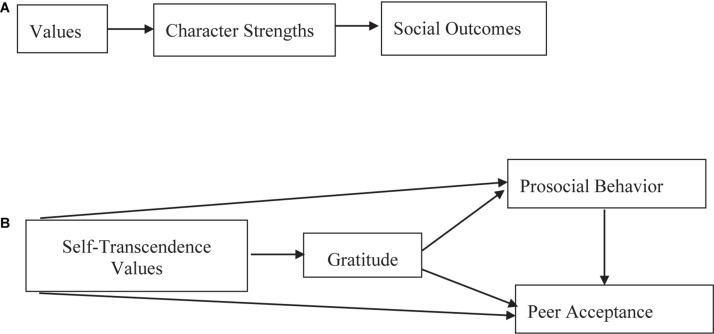
**(A)** General theoretical model. **(B)**The research model.

These compelling theoretical ideas portraying character strengths as psychological pathways for pursuing moral values have attracted surprisingly limited empirical examination (although their connections with positive behavior and social outcomes have been demonstrated; e.g., Niemiec, 2013). This may be partly because the initial categorization linking specific character strengths with specific virtues, suggested by Peterson and Seligman (2004), has been subjected to criticism about the theoretical structure and connections among the strengths and between strengths and virtues, and has received limited empirical support (e.g., Kristjánsson, 2013; McGrath, 2019; Miller, 2019; Snow, 2019; Stichter and Saunders, 2019). Another reason may be that virtues were typically computed as aggregated character strengths measures, as detailed below (e.g., McGrath, 2014, 2015). In our article, we suggest a possible way to fill this void by bringing together two theoretical frameworks, one for human values (Schwartz, 1994) and the other for character strengths (Peterson and Seligman, 2004), and examining character strengths as potential pathways for behavioral manifestation of values as defined by the human values theory, linking them to positive social outcomes (Figure 1A).

The character strengths and virtues framework (Peterson and Seligman, 2004) is different from the human values theory (Schwartz, 1994, 2012), and the meaning of *values* in the two theories is different (as detailed below). However, as also noted by Peterson and Seligman (2004), there are notable connections between the theories, and certain constructs seem to have parallel elements. In what follows, we delineate commonalities and differences and suggest that connections between the two theories can provide a valuable route to an external, empirical examination of character strengths as values in action. We then provide an initial example of this kind of empirical exploration, in a humble exploratory case study of one character strength – gratitude – hypothesized to provide a pathway from self-transcendence values with two frequently studied positive social outcomes, prosocial behavior, and peer acceptance (Schwartz, 2010; Cillessen and Bellmore, 2011). Based on the theoretical model, this idea is examined in two samples of adolescents.

### Character Strengths

Character strengths have been widely researched over the past decade (e.g., Harzer, 2016), and their endorsement and use are consistently associated with increased well-being and functioning (e.g., Lavy et al., 2014; Littman-Ovadia et al., 2016; Lavy and Littman-Ovadia, 2017). As noted above, they have been defined as “values in action” (VIA; e.g., Park and Peterson, 2006), representing the psychological manifestations of moral values, reflected in thoughts, feelings, and behavior. As such, they represent “the psychological processes or mechanisms that define the virtues” (Park and Peterson, 2006; p. 893), with virtues considered basic values appreciated across cultures (Peterson and Seligman, 2004; Park and Peterson, 2006). Thus, character strengths are theorized to create the pathway through which core values (or “core virtues” in Peterson and Seligman’s words) become mundane behaviors, leading to positive social outcomes.

Although notable research has explored the associations of character strengths with the anticipated end result of practicing noble values – personal and social well-being and functioning (e.g., Littman-Ovadia and Lavy, 2012; Harzer, 2016; Lavy et al., 2016; Littman-Ovadia and Lavy, 2016) – more limited empirical evidence links character strengths with valued social outcomes, and this evidence usually focuses on a few specific character strengths (Niemiec, 2013). Empirical research linking character strengths with values is equally scarce and is typically restricted to examinations of the hierarchical structure of character strengths and virtues (each virtue is thought to reflect basic values, which can be manifested *via* certain character strengths). Such examinations have questioned the initial theoretical structure suggested by Peterson and Seligman (2004), as various studies revealed a factorial structure of character strengths which was different than expected [for a review and analysis, see McGrath (2014, 2015)]. More important for present purposes, when examining links of virtues/values and character strengths, researchers have typically remained within the character strengths framework only. We sought to broaden the understanding of character strengths’ relations with values by including another well-known framework of human values, posited by Schwartz (1994).

### Proposed Theoretical Integration of Character Strength and Virtues and Human Values Theories

In his seminal theory of human values, Schwartz (1992, 2012) defined values as representations of desirable goals and important broad motivators of behavior. Thus, Schwartz’s values are not identical to Peterson and Seligman’s (2004) strengths and virtues: while Schwartz’s working definition concerns what people value or think is important in their lives, character strengths and virtues are concerned with people’s characteristics. Moreover, Peterson and Seligman’s (2004) character strengths and virtues have moral valence. They are based on what are considered noble values, ones that people should appreciate. Schwartz’s (1994, 2012) human values represent what people value in a non-judgmental, descriptive (not prescriptive) attitude, making his theory, in essence, a-moral.

There are four types of higher-order values, each representing a broad motivational goal: self-transcendence, openness-to-change, self-enhancement, and conservation (Schwartz, 1992, 1994). This values structure has been found in 70 countries (e.g., Schwartz and Rubel, 2005), and research has consistently demonstrated associations of the four value types with personality (Parks-Leduc et al., 2015), attitudes (Boer and Fischer, 2013), and behaviors (Bardi and Schwartz, 2003; Benish-Weisman, 2015, 2019).

We suggest that each higher-order value will be associated with certain virtues or virtues that comprise character strengths reflecting the personal characteristics required to attain the goals related to this value (Table 1). This is different from Peterson and Seligman’s (2004) approach to the connection between the two theories, as they expressed an interest in comparing the measures of character strengths (not virtues) to those of specific values (not higher-order values). Such connections may indeed be more accurate, especially as the hierarchical structure of the VIA virtues has not gained much empirical support (as mentioned above; e.g., McGrath, 2015). However, we chose to explore, in our theoretical overview, the links between the higher-order levels in both theories (i.e., virtues and higher-order values): We feel that linking more specific levels may be premature at this initial stage of mapping the links between the theories and require more information (including empirical evidence) about such connections. Furthermore, as each virtue is hypothesized to be manifested *via* a few character strengths, it is sensible to examine whether the character strengths related to a certain virtue indeed “operate” as pathways from the higher-order values paralleled with this virtue and the expected social outcomes (Figure 1A).

**TABLE 1:** An initial suggestion for corresponding values and virtues.

**Schwartz’s higher-order values (and the values related to them)**	**VIA virtues (and the strengths related to them)**	
**Self-transcendence** reflects a concern for the welfare and interests of others (universalism and benevolence).	**Transcendence** reflects the connection to something “higher,” something larger than ourselves, which can provide a sense of purpose or meaning (gratitude*, hope, humor, spirituality*, appreciation of beauty, and excellence*).	**Humanity** reflects feelings and values of basic love and companionship with all human beings (love, kindness, and social intelligence).
**Conservation** is concerned with order, self-restriction, preservation of the past, and resistance to change (security, conformity, and tradition).	**Temperance** (or moderation) is related to self-management, and conservation of social harmony and resources (forgiveness∼, modesty, prudence, and self-regulation).	**Justice** is about the connections with the community or group in different ways and situations (fairness*, leadership∼*, and teamwork/citizenship).
**Openness to change** is related to independence of thought, action, and feelings and readiness for change (self-direction and stimulation).	**Courage** focuses on strength of will, and pursuing one’s beliefs and goals even in the face of adversities (bravery, persistence∼*, honesty, and zest).	
**Self enhancement** reflects a focus on pursuing one’s own interests, success, and dominance over others (power and achievement).		
		**Wisdom** is about good judgment, based on profound knowledge and understanding (creativity*, curiosity, love of learning*, judgment, and perspective*).

∼The Human Values Theory claims the strength is related to a different value. Specifically, it suggests forgiveness is related to self-transcendence values, and leadership is related to self-enhancement values.*Peterson and Seligman (2004) claim the strength is related to a different value. Specifically, they suggest appreciation of beauty and excellence corresponds with hedonism; gratitude and spirituality are related to conservation values (i.e., security and tradition, respectively); fairness and perspective are related to self-transcendence values (i.e., universalism); creativity, leadership, and persistence are related to self-enhancement values (i.e., corresponding with self-direction, power, and achievement values, respectively); curiosity and love of learning are related to openness to change values (i.e., stimulation).

We followed Peterson and Seligman’s (2004) suggestion that Schwartz’s values assessment is “not identical with the measures of strengths we have developed. It measures what people value, not their traits or habitual actions” (p. 76). Following this line of thought, we explored the associations of Schwartz’s (2012) higher-order values with Peterson and Seligman’s (2004) typology of virtues, arguing that if character strengths are indeed “values in action,” they will be related to Schwartz’s (2012) values. Furthermore, in these cases, character strengths will serve as the psychological mechanism promoting the behaviors embodying these values.

#### Connecting Specific Human Values With VIA Virtues

Before we move to the specific initial examination, we would like to offer an integrative framework. Our proposed theoretical connection is summarized in Table 1. We believe that the most salient connection of values and virtues is that between the higher-order *values of self-transcendence* stressing concern for the well-being and interests of others (Schwartz, 1994) and the VIA *virtue of transcendence* focusing on the connection to something larger than ourselves and looking above our own needs to engender a sense of purpose or meaning (Peterson and Seligman, 2004). Connecting to something above ourselves can be translated into taking care of others, as in self-transcendence values. Another core virtue which can be perceived as closely related to the higher-order value of self-transcendence is *humanity* – related to basic love of and companionship with all fellow humans (Peterson and Seligman, 2004). Linking VIA virtues to self-transcendence values is relatively intuitive, as these values focus on others/the universe, and can thus be considered moral (e.g., Han, 2019- about other-focused values and morality).

Higher-order *conservation values*, reflecting concerns for order, self-restriction, preservation of the past, and social harmony (Schwartz, 2012, p. 8), correspond with core aspects of the VIA virtues of *justice*, focusing on connecting with the community or group, and *temperance*, focusing on self-management and the maintenance of harmony with others.

The higher-order *openness-to-change values*, comprising values related to independent thought, action, and feelings (Schwartz, 2012, p. 8), correspond with certain aspects of the VIA virtue of *courage* and its concern with pursuing one’s will in the face of adversity. However, this connection is more complex, as courage may be used to pursue values not closely connected to change and may even contradict it. This is also revealed in the strengths related to courage. It is possible to see how openness-to-change is related to two of these strengths – bravery and zest. However, the other two strengths related to courage, perseverance, and honesty seem more loosely connected to openness-to-change. Thus, in this case, a more nuanced connection of specific strengths with Schwartz’s (2012) values may be more helpful.

In a similar vein, it is difficult to link the VIA virtue of *wisdom* to a specific higher-order value of Schwartz’s (1994) theory. Wisdom seems to be an inclusive virtue, more loosely connected to a certain set of beliefs; it represents an advanced state of personal knowledge and understanding, stemming from highly developed perceptions and interpretive abilities and courageous actions to pursue it (Peterson and Seligman, 2004). Interestingly, most character strengths categorized under the wisdom virtue can be connected to other higher-order values (e.g., openness to change: creativity, curiosity, love of learning, perspective).

From the human values perspective, it is difficult to find a virtue that corresponds with *self-enhancement* values. Although certain aspects of courage and temperance can be related to the pursuit of personal success (e.g., perseverance and self-regulation), the moral valence of the VIA virtues limits their focus on self-enhancement and, in essence, gives more attention to other-oriented values, concerned with the good of others, and society. As noted above, while Schwartz’s (1994) values theory is non-judgmental (i.e., a-moral), virtues are defined as “dispositions to behave in moral ways” (Park and Peterson, 2006, p. 895), emphasizing a universal moral valence.

#### Interim Summary

Despite the different focuses, Schwartz’s human values conceptualization and measurement can be helpful in providing a broader theoretical perspective and alternative tools for examining character strengths as pathways from values to behaviors and social outcomes. Like virtues, Schwartz’s higher-order values are perceived as basic constructs reflecting desirable ideas/goals and guiding desirable behavior. And as demonstrated above (and in Table 1), there are multiple links between higher-order values and virtues. To the best of our knowledge, although the link of Schwartz’s (1994) values with character strengths in pursuit of predicting behavior has been suggested (Crossan et al., 2013), no studies have systematically examined associations of character strengths with other classifications of values.

In this research, we began to examine the theoretical model in which character strengths are theorized to be the pathways linking values/virtues (which are suspected to be paralleled, as mentioned above) with social outcomes. This examination is complex, especially because the various 24 character strengths (Peterson and Seligman, 2004) provide paths from the four higher-order values (Schwartz, 1994) to a host of social behaviors and outcomes. Thus, we conducted only an initial examination of one case in the theoretical model (Figure 1B), focusing on the more evident and relatively clear conceptual connections. Specifically, we focused on one character strength – gratitude – as a possible pathway from self-transcendence values to two positive social outcomes (Schwartz, 2010; Cillessen and Bellmore, 2011): prosocial behavior and peer acceptance.

### Gratitude, Self-Transcendence Values, and Positive Social Outcomes

Gratitude, one of the 24 character strengths, has been studied by psychologists, philosophers, and theologists and has several definitions (Gulliford et al., 2013). Focusing on Peterson and Seligman’s (2004) definition, we propose that it reflects a person’s awareness and thankfulness for a good thing that has happened and/or the devotion of time to express this awareness and is related to the virtue of transcendence, because it can enable individuals to connect to the “larger universe” and give meaning to their lives (p. 519). The acknowledgment of goodness bestowed upon them is expected to connect people directly with goodness (in its diverse expressions) and the notion that we have benefited from someone else actions, resulting in feelings of grace, is considered a transcendent emotion (Peterson and Seligman, 2004, p. 524).

#### Gratitude and Values

Empirical research provides some support for the categorization of gratitude as a strength of transcendence, while showing its associations with altruistic values (e.g., Romani et al., 2013). Thus, we suggest gratitude is positively related to self-transcendence values (Schwartz, 1992). It should be acknowledged that Peterson and Seligman (2004, p. 74) argue that gratitude corresponds with the value of security, because it may be related to nurturing and strengthening close relationships that provide security. Although gratitude may indeed serve the need for security (and the more basic value of conservation), we rely on its adherence to the initial definition of self-transcendence and on recent literature (accumulated after 2004) to suggest it may be more closely related to self-transcendence values.

#### Gratitude as a Pathway From Self-Transcendence Values to Positive Social Outcomes

We further suggest that gratitude will provide a pathway from self-transcendence values to prosocial behavior and peer acceptance – two social outcomes with a far-reaching impact on human lives (Prinstein and La Greca, 2004), because they help preserve the social fabric required for human existence and thriving. Although all higher-order values defined by Schwartz (1994) have the potential to contribute to individuals and societies, the values which are theoretically most closely connected to prosocial behavior and positive social outcomes are self-transcendence values (Schwartz, 2010; Arieli et al., 2014). These values emphasize concern for the well-being and interests of others, and their positive association with and effect on prosocial behavior have been established in a laboratory setting (e.g., Maio et al., 2009; Arieli et al., 2014). However, knowledge of these relations in natural settings and of the personality mechanisms through which they operate (e.g., gratitude or other character strengths) is scarcer (e.g., Benish-Weisman et al., 2019), and their examination could help explain the antecedents of these desired social behaviors.

Our decision to also examine peer acceptance was based on the compelling evidence that values are related to both behavior and social adjustment (Sagiv and Schwartz, 2000). We argue that transcendence values may be related to a specific kind of social adjustment – acceptance by the peer group – because they entail a focus on others and concern for the social environment. We further proposed that gratitude may pave the path from self-transcendence values to peer acceptance, because one of the ostensible functions of gratitude is to build and preserve relationships by encouraging reciprocity of the “grace” individuals receive from others, thus promoting prosocial behavior and partnerships (Emmons and McCullough, 2004; Bartlett and DeSteno, 2006). Empirical studies have demonstrated that gratitude is linked with and affects not only prosocial behavior, but also positive relationships and social integration (McCullough et al., 2002; Bartlett and DeSteno, 2006; Algoe et al., 2008; Froh et al., 2010; Wood et al., 2010).

In sum, the integrative research model (Figure 1B) suggests self-transcendence values give rise to gratitude, which, in turn, promotes prosocial behavior and peer acceptance:

*H1: Gratitude will provide a pathway from self-transcendence values to prosocial behavior*.
H2: Gratitude will provide a pathway from self-transcendence values with peer acceptance.

### The Present Study

We examined the model in two samples of adolescents. We focused on adolescents because values and character strengths are thought to develop during adolescence (Weber et al., 2013; Daniel and Benish-Weisman, 2019), and social behavior and outcomes are especially important (Parker et al., 2006). Initial examination of the research model was conducted in a pilot sample comprising mostly Jewish-Israeli 9th graders. Then, we replicated the findings in a larger, more diverse sample. Acknowledging the importance of cultural context in examining the application of moral values/virtues (e.g., Han, 2019; Snow, 2019), and the notable differences between Jews and Arabs living in Israel in terms of values, behavior, and social outcomes (e.g., Daniel et al., 2014), the main sample comprised both Jewish and Arab 11th graders.

## Method

### Participants

Sample size was determined using power analysis in G^∗^Power 3.1. Based on correlations extracted from a data set collected for a previous study (Knafo-Noam, Unpublished data set, also used in Abramson et al., 2018), the association between prosocial behavior and self-transcendence values was 0.25. Assuming one-tailed α values of 0.05, the required sample size was 168. The pilot sample comprised 161 students (53.4% women) in the 9th grade in a Jewish school in Israel. Most were Jews born in Israel (78.1%) or Jews born in Russia (19.2%), with a few other ethnicities (2.7%). The second, main sample comprised 344 (51% girls) 11th grade students from four high schools in Israel, including Jews born in Israel (34.5%), Arabs born in Israel (33%), and Jews born in Russia (31%).

### Measures

*Self-transcendence values* were measured with the Portrait Values Questionnaire (PVQ; Schwartz et al., 2001), previously found suitable for use with children and adolescents (Schwartz et al., 2001; Knafo et al., 2008). The PVQ includes four sub-scales assessing the four higher-order values. The self-transcendence subscale includes short verbal descriptions of 10 people (matched to the respondent’s gender) indicating the importance of caring for the welfare and interests of others (e.g., “It’s very important for her to help the people around her. She wants to care for their well-being”). For each description, participants rate their similarity to the person described, on a 6-point scale, ranging from 1 (not like me at all) to 6 (very much like me). Respondents’ own values are inferred from their self-reported similarity to the described people. As a standard procedure when using the PVQ, we controlled for response tendency by centering each participant’s responses around his or her average response to all questions on the scale (Bardi et al., 2014). The scale’s internal reliability was good in both the pilot and main samples (α’s = 0.82 and 0.85, respectively).

*Gratitude* was assessed with the first five items of the Gratitude Questionnaire (GQ-6; McCullough et al., 2002; Froh et al., 2011). The last item was omitted because low loadings have consistently been found in previous studies, especially in youth (Froh et al., 2011). Participants’ agreement with each item (e.g., “I have so much in life to be thankful for”) was rated on a scale ranging from 1 (*strongly disagree*) to 7 (*strongly agree*). The scale’s reliability was satisfactory (α = 0.74 and 0.78 for the pilot and main samples, respectively).

*Prosocial behavior* was assessed by peer nomination (Ungvary et al., 2018). The inventory included three questions tapping prosocial behavior (e.g., “Who cooperates?”), and participants marked, on a list of their classmates, the names of those whose behaviors fit each of the given descriptions. Each participant’s score on each item was computed by dividing the number of nominations he or she received by the total number of classmates who could have nominated him or her for that item. The final scores for each item were standardized within all the participating students within each class. The scale’s reliabilities are α = 0.83 and 0.64 in the pilot and main samples, respectively.

*Peer acceptance* was also assessed by peer nomination. The inventory included four items tapping social acceptance (e.g., “Who is liked by the other children?”), and participants marked, on a list of classmates, those that fit the descriptions. Items were scored as described above (prosocial behavior assessment). The scale’s reliability was good (α = 0.89 and 0.78 for the pilot and main samples, respectively).

### Procedure

Consent forms were sent to parents of students in participating schools, with over 95% approval rate. Trained research assistants distributed the questionnaires to the participating children during a class session. As a token of gratitude for their participation, students received small, attractive incentives (e.g., pencils). The University of Haifa and the Israeli Ministry of Education ethical review boards approved the study.

## Results

**FIGURE 2:**
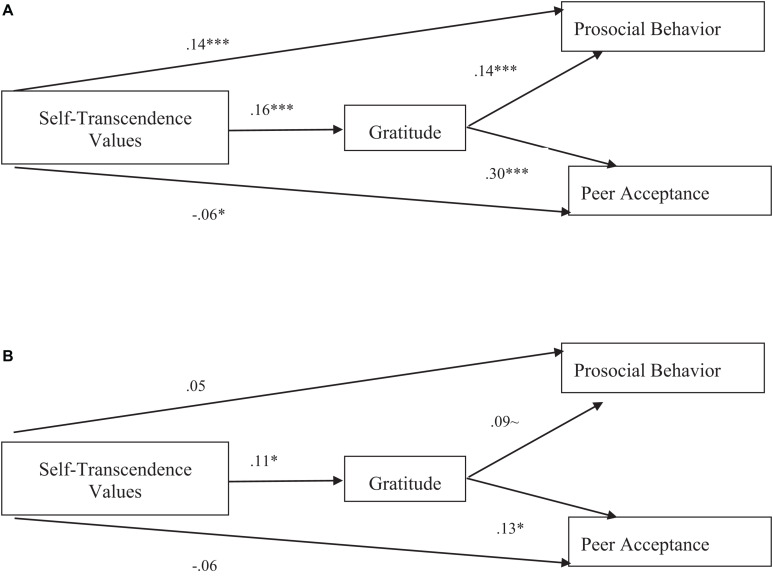
**(A)** Standardized estimates of SEM saturated model linking self-transcendence values, gratitude, prosocial behavior, and peer acceptance in 9th graders (pilot sample). *P < 0.05; ***P < 0.001. All direct and indirect effects were significant. The standardized indirect effect of self-transcendence values was 0.023, *P* < 0.001 (SE = 0.004; 95% CI = 0.015, 0.033) on prosocial behavior, and was 0.048, *P* < 0.001 (SE = 0.007; 95% CI = 0.035, 0.062) on peer acceptance, suggesting that both indirect effects were significant. **(B)** Standardized estimates of SEM saturated model linking self-transcendence values, gratitude, prosocial behavior and peer acceptance in 11th graders (main sample). Notes: *P < 0.05; ∼*P* = 0.08. The standardized indirect effect of self-transcendence values was 0.11, *P* = 0.058 (SE = 0.008; 95% CI = 0.000, 0.033) on prosocial behavior, and was 0.015, *P* < 0.05 (SE = 0.009; 95% CI = 0.002, 0.039) on peer acceptance, suggesting that the first indirect effect was marginally significant, and the second was significant. All error terms were omitted in the figures, as well as the covariance between the dependent variables, to enhance simplicity and comprehension; ethnicity and gender were controlled.

Table 2 presents the variables’ means, standard deviations, and zero-order correlations. The research model (Figure 1B) was examined using structural equation modeling (SEM) in the AMOS statistical package, while including the covariance between the dependent variables, and controlling for gender (and also ethnicity, in the main sample). For the pilot sample, the measurement model showed good fit with the data (TLI = 0.91, CFI = 0.92, RMSEA = 0.06). The items’ loadings were acceptable: The self-transcendence items loadings were 0.42–0.69 (with one exception 0.28), and the gratitude, prosocial behavior, and peer acceptance items had loadings of 0.44–0.82, 0.67–0.89, and 0.82–0.90, respectively. In the research model (path analysis), all direct and indirect paths were significant (see details in Figure 2A), and thus, both research hypotheses (H1 and H2) were supported.

**TABLE 2:** Means, standard deviations, and correlations of sample 1 and 2 variables.

	**Means**	**SD**	**Self-transcendence values**	**Gratitude**	**Prosocial behavior**
**Sample 1**					
Self-transcendence values	4.23	0.46			
Gratitude	5.38	1.04	0.12		
Prosocial behavior	0.03	0.80	0.18*	0.17^†^	
Peer acceptance	0.06	0.77	0.02	0.28***	0.31***
**Sample 2**					
Self-transcendence values	4.15	0.50			
Gratitude	4.96	1.37	0.13**		
Prosocial behavior	0.15	0.81	0.06	0.10^†^	
Peer acceptance	0.12	0.80	–0.03	0.14**	0.52***

^†^P < 0.1.; *P < 0.05; **P < 0.01; ***P < 0.001.

In the main sample, again, the measurement model showed a good fit to the data (TLI = 0.90; CFI = 0.92; RMSEA = 0.60). The self-transcendence items loadings were 0.51–0.75 (with one exception −0.30). The gratitude, prosocial behavior, and peer acceptance items had loadings of 0.64–0.82, 0.72–0.83, and 0.67–0.83, respectively. In the research model (path analysis), the direct effects of self-transcendence values on gratitude and of gratitude on peer acceptance were significant, as well as the indirect effect of self-transcendence values on peer acceptance *via* gratitude (supporting H1). The direct effect of gratitude on prosocial behavior and the indirect effect of self-transcendence values on prosocial behavior *via* gratitude were marginally significant (see details in Figure 2B), providing marginal support for H2.

## Discussion

The paper introduces a framework for exploring character strengths’ role as “values in action,” linking Peterson and Seligman’s (2004) theoretical framework with that of Schwartz (1994, 2012). It suggests that the VIA character strengths (Peterson and Seligman, 2004) may provide a path from higher-order values (as defined by Schwartz, 1994) to behaviors and social outcomes. Thus, character strengths may serve as psychological mechanisms driving the pursuit of these values in life. We show how this connection between the theories can be examined in an initial example of a case study of gratitude.

The empirical study focused on a higher-order value (self-transcendence) which has a relatively salient connection with the VIA virtue transcendence. It examined one pathway from this value to social outcomes (prosocial behavior and peer acceptance) – *via* gratitude, a character strength thought to present a psychological manifestation of transcendence. We examined this theoretical model in two samples. The results generally supported the model, suggesting that gratitude may serve as a pathway from self-transcendence values to prosocial behavior and peer acceptance. However, in the main sample, the indirect path to prosocial behavior *via* gratitude was only marginally significant, suggesting that other factors may be involved and that more research is needed.

The findings provide initial empirical evidence that character strengths may serve as psychological mechanisms linking values with behavior, as theorized (e.g., Park and Peterson, 2006). They shed light on how the application of values, which are broad and abstract, can be encouraged, as character strengths can be cultivated through practice (Peterson and Seligman, 2004; Quinlan et al., 2012). Furthermore, because character strengths’ use and development depend (at least to some extent) on an individual’s social, organizational, and familial contexts (e.g., Harzer and Ruch, 2013; Lavy et al., 2017; Lavy, 2020), we may be able to enhance their use and promote positive social behavior by structuring environments (e.g., workplaces, schools) in ways that encourage it. For example, self-transcendence may be practiced in classes (or organizations) by encouraging gratitude expressions. However, as in the second sample, the indirect path to prosocial behavior *via* gratitude was only marginally significant, these findings should be interpreted with caution, while acknowledging that additional factors may heavily influence social behavior and outcomes, and other processes (e.g., developmental and cultural) may affect the moderating role of character strengths.

We also had other unexpected findings not related to the main hypotheses: the zero-order correlations of self-transcendence values with gratitude (pilot sample) and with prosocial behavior (main sample) were not significant (Table 2). These findings may be due to demographic factors that were controlled in the subsequent analyses (i.e., gender and culture) and may be worthy of further investigation in light of the cultural characteristics of the two samples: The Jewish population is characterized more by Western and individualistic values, but also prize family and communal values (Mayseless and Salomon, 2003; Mayseless and Scharf, 2003). The Arabic population is considered in transition, but traditionally endorses more conservative and collectivistic values (Lapidot-Lefler and Hosri, 2016).

The research findings should be considered in light of its limitations; our cross-sectional design did not allow inference of causality. Our analysis was based on limited questionnaires and peer assessment, and the samples included only adolescents from only two cultures in one country. Although the design included relatively powerful measurements of behavior and social outcomes of peer nominations (which are less inclined to social desirability effects), further exploration of concrete behaviors can be helpful, as well as a longitudinal study. Furthermore, although the theoretical research framework generally proposes that the 24 character strengths serve as pathways from values to social outcomes, the present research provides a very humble empirical examination of this idea, in a specific case – of one set of values, one character strength, and two social outcomes. A more thorough examination of other character strengths as pathways from values to social outcomes is needed in order to ascertain its validity. Such examination can also help map the connections of human values with character strengths.

Despite the limitations of the research, our findings offer initial evidence for potential links of Schwartz’s (1992) human values theory with the VIA framework, thus deepening our understanding of social behavior. As no studies to date have systematically examined associations of character strengths with other classifications of values, we hope this research will inspire further empirical research that openly links character strengths to other theoretical frameworks.

## Data Availability Statement

The raw data supporting the conclusions of this article will be made available by the authors, without undue reservation.

## Ethics Statement

The studies involving human participants were reviewed and approved by the University of Haifa, Faculty of Education Ethics Committee and the Chief Scientist of the Israeli Ministry of Education. Written informed consent from the participants’ legal guardian/next of kin was not required to participate in this study in accordance with the national legislation and the institutional requirements.

## Author Contributions

MB-W was responsible for data collection. SL wrote an initial draft, on which MB-W provided the meaningful comments and changes, and both authors continued to work together on the manuscript until finalized. Both authors jointly developed the theoretical framework and research design and conducted the analysis together.

## Conflict of Interest

The authors declare that the research was conducted in the absence of any commercial or financial relationships that could be construed as a potential conflict of interest.
